# A case of enormous retroperitoneal liposarcoma with prolapse from the left inguinal canal following hernia repair

**DOI:** 10.1186/s40792-024-01891-0

**Published:** 2024-04-24

**Authors:** Sho Ueda, Takuya Saito, Yasuyuki Fukami, Shunichiro Komatsu, Kenitiro Kaneko, Tsuyoshi Sano

**Affiliations:** Department of Gastoroenterological Surgery, Aichi Medical University, 1-1 Yazakokarimata, Nagakute, Aichi 480-1195 Japan

**Keywords:** Sarcoma, Retroperitoneal, Inguinal hernia

## Abstract

**Background:**

Liposarcomas represent ~9.8–16% of soft tissue sarcomas, with the extremities and retroperitoneum being the primary sites of occurrence. While liposarcoma in the inguinal region is uncommon, few reported cases originate from the retroperitoneum and protrude into the scrotum through the inguinal canal. Here, we present a case of a retroperitoneal liposarcoma with prolapse from the left inguinal canal into the scrotum following hernia repair with a mesh plug.

**Case presentation:**

A 55-year-old male patient underwent a CT scan for a suspected recurrent inguinal hernia, which revealed a sizeable adipose-dense tumor by the left kidney extruded through the left inguinal canal surrounding the scrotum. The patient had undergone mesh plug repair for a left inguinal hernia at another hospital one year ago and noticed ipsilateral inguinal swelling after the hernia repair. The patient was referred to our hospital. The tumor resection was completed with combined resection of potentially involved organs: left side colon, left kidney, and left adrenal gland. Also, complete excision of the tumor was accomplished through surgical resection of the posterior wall of the inguinal canal, the mesh plug, and the tumor extending into the scrotum. Given the nearly complete absence of the inguinal canal's posterior wall and the anterior wall’s torn state, sutures were employed to close the external obturator tenosynovitis. Additionally, the inguinal ligament was closed using a tension-free incision technique. Only a mesh was subsequently placed. The resected tumor measured 47 × 30 × 15 cm and 7.5 kg in weight. After surgical resection, a retroperitoneal liposarcoma diagnosis was established. After 2 years and 6 months following the surgical resection, no recurrence has been observed for either liposarcoma or inguinal hernia.

**Conclusion:**

The previous inguinal hernia in this case must be a prolapse of retroperitoneal liposarcoma. Thus, it is recommended to conduct a preoperative examination, which should include a CT scan, since the presence of a fatty mass within the hernia may indicate the presence of a retroperitoneal liposarcoma. Even if a preoperative diagnosis cannot be made, a long-term prognosis can be expected if the retroperitoneal liposarcoma can be completely resected at reoperation.

## Background

Spermatic cord lipomas are often identified intraoperatively during inguinal hernia repair procedures. However, liposarcomas originating from the spermatic cord are relatively uncommon. Liposarcomas constitute ~9.8–16% of soft tissue sarcomas [1, 2] and are frequently found in the extremities and retroperitoneum [3]. Instances of retroperitoneal liposarcomas extending from the inguinal canal are exceedingly uncommon. This report details a case involving an exceptionally enormous retroperitoneal liposarcoma that had descended from the left inguinal canal into the scrotum. Notably, no recurrence was observed following surgical intervention.

## Case presentation

**Fig. 1:**
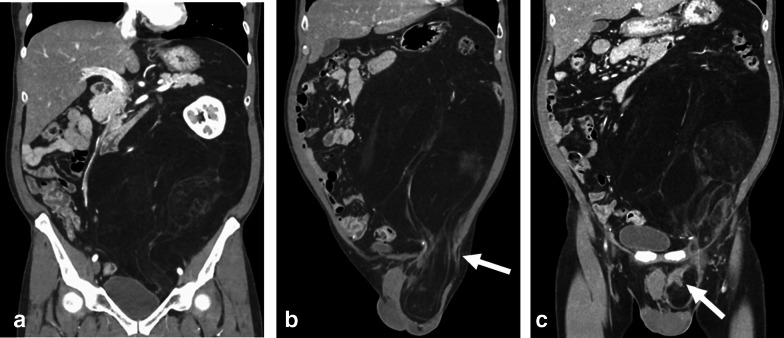
**a**. Contrast-enhanced CT imaging exhibited a substantial adipose-dense tumor originating from the caudal aspect of the left kidney. **b**. The tumor has been extending through the left inguinal canal, and reaching the scrotum (indicated by arrows). **c**. Notably, the mesh plug that had been implanted one year earlier was visibly protruding due to the presence of the tumor (indicated by arrowheads)

**Fig. 2:**
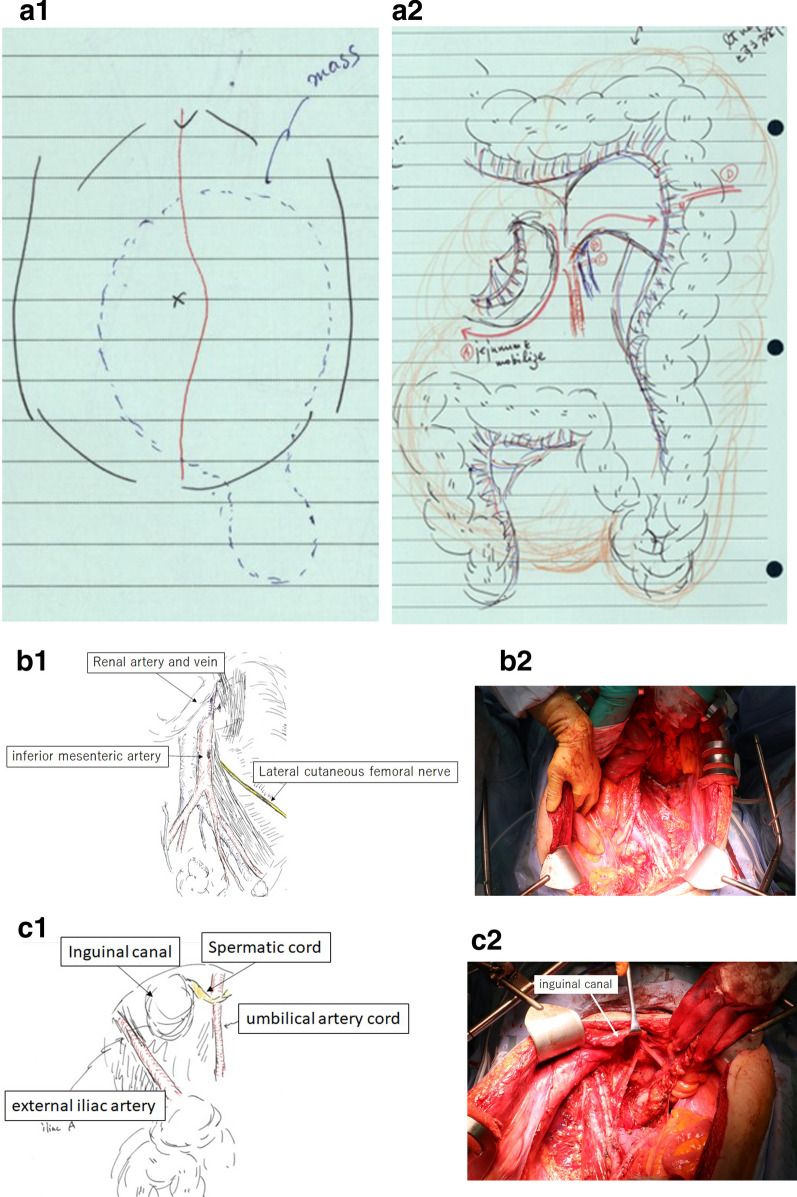
**a1,2**. The tumor was visibly exerting pressure on the left colon and left kidney. **b1,2**. Given the extensive involvement, a combined resection of the left colon and left kidney was undertaken. **c1,2**. The tumor was entirely excised through the resection of the posterior wall of the inguinal canal, the mesh plug, and the tumor's extension into the scrotum

**Fig. 3:**
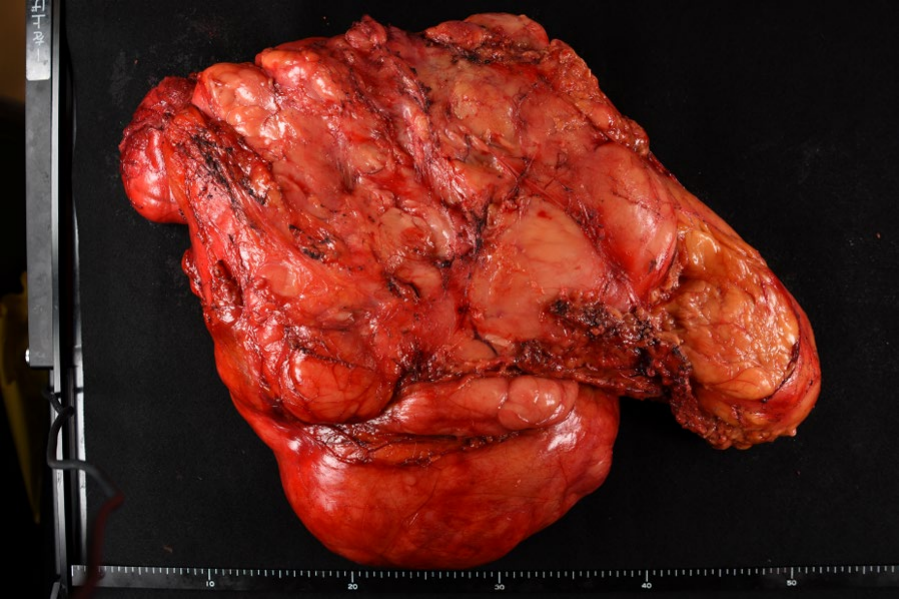
The resected tumor measured 47 × 30 × 15 cm and 7.5 kg in weight

**Fig. 4:**
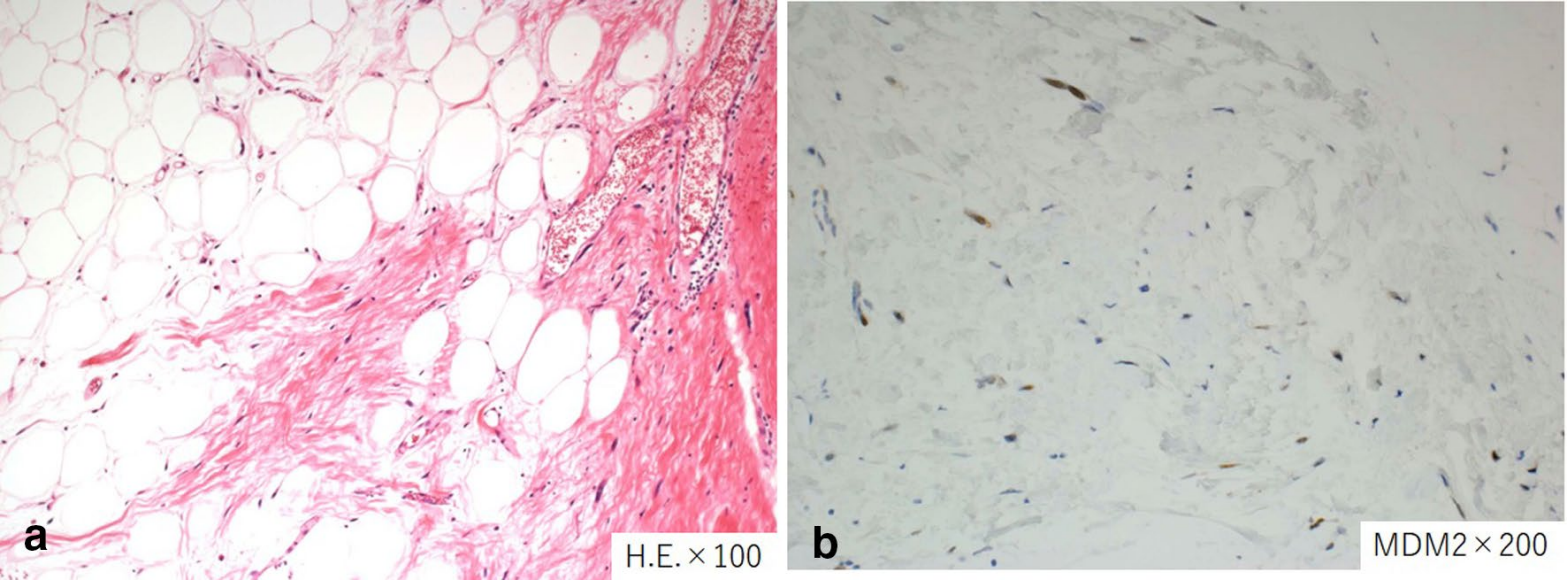
Pathological examination confirmed the tumor's identity as a well-differentiated liposarcoma (**a**. H.E.×100 **b**. MDM2×200)

A 55-year-old male patient presented to our hospital with a significant bulge in the left inguinal region; he had undergone mesh plug repair of a left inguinal hernia at another hospital one year ago. Contrast-enhanced computed tomography (CT) revealed an enormous fat-dense tumor originating from the caudal side of the left kidney and extending through the left inguinal canal, eventually reaching the scrotum (Fig. 1a, b). The implanted mesh plug was bulging due to the presence of the tumor (Fig. 1c). The diagnosis was suspected of an enormous retroperitoneal liposarcoma that had prolapsed through the inguinal canal and extended into the scrotum. Surgery was executed to resect the retroperitoneal liposarcoma, accompanied by resection of the left-sided colon and left kidney (Fig. 2a). Concurrently, repair of the left inguinal hernia was undertaken. The tumor showed significant involvement of the left colon and left kidney, necessitating simultaneous resection of both organs. Complete tumor excision was accomplished by removing the mesh plug, which had become integrated with the tumor, extending from the posterior wall of the inguinal canal to the scrotum (Fig. 2b, c). The posterior wall of the inguinal canal displayed significant structural compromise, while the anterior wall showed evidence of tearing. Meticulous suturing of the external oblique aponeurosis to the inguinal ligament was performed, along with a reflexing incision to optimize closure. Only mesh was employed for the repair. The total operative duration was 7 h and 47 min, accompanied by a blood loss of 1489 ml. The resected tumor measured 47 × 30 × 15 cm and 7.5 kg in weight (Fig. 3). Pathological examination confirmed the tumor's identity as a well-differentiated liposarcoma (Fig. 4).

The patient was subsequently discharged on postoperative day 33. Impressively, no recurrence has been observed over 2 years and 6 months post-surgery for either liposarcoma or inguinal hernia.

## Discussion

We reviewed reported cases of retroperitoneal liposarcoma escaping the inguinal canal, including one autopsy case, and made two findings. The first is the condition under which retroperitoneal liposarcomas can escape from the inguinal canal. The second is how to deal with a retroperitoneal liposarcoma that has prolapsed from the inguinal canal and has been operated on as an inguinal hernia.

Retroperitoneal liposarcoma prolapsing through the inguinal canal remains an infrequent phenomenon. A PubMed search utilizing the keywords "retroperitoneal liposarcoma" and "inguinal" yielded 14 case reports. Inclusive of our case, Table 1 compiles 15 such instances [4–16]. Notably, all identified cases involved male patients aged between 40 and 86 years, with a mean age of 58.9. The dimensions of the tumors exhibited significant variability, spanning from 17 to 70 cm, with an average of 36.2 cm. Laterality presented no notable asymmetry, as eight cases occurred on the right side and seven on the left. Postoperative histopathological evaluations revealed that well-differentiated liposarcomas characterized 12 cases, while the remaining three exhibited pleomorphic features. Retroperitoneal liposarcomas are recognized for their elevated malignant potential, with Enterline et al. reporting distant metastases in 31% of liposarcoma patients [17]. In contrast, well-differentiated liposarcomas inherently possess lower malignant potential [18]. The emergence of retroperitoneal liposarcomas protruding through the inguinal canal appears contingent on two conditions: the presence of an indirect inguinal hernia and a low-grade malignant tumor capable of asymptomatic growth until it extends from the inguinal canal.

**Table 1:** Case reports of retroperitoneal liposarcoma prolapse from the inguinal canal

No.	Author	Year	Age/sex	Size (cm)	Weighed (g)	Right/left	Type
1	Noguchi [4]	2001	60/M	24	N/A	Right	Pleomorphic
2	Mizuno [5]	2006	53/M	45	7510	Left	Well-differentiated
3	Baldassarre [6]	2007	69/M	N/A	N/A	Left	Well-differentiated
4	Ghimire [7]	2011	53/M	28	N/A	Right	Well-differentiated
5	Bhandarwar [8]	2011	40/M	47	12,700	Right	Well-differentiated
6	Leão [9]	2011	86/M	30	7500	Right	Well-differentiated
7	McKinley [10]	2013	63/M	17	N/A	Right	Well-differentiated
8	Tardu [11]	2016	53/M	48	N/A	Left	Pleomorphic
9	Fiaschetti [12]	2017	64/M	21	N/A	Left	Well-differentiated
10	Fiaschetti [12]	2017	67/M	24	N/A	Left	Well-differentiated
11	Lechner [13]	2019	57/M	46	1261	Right	Well-differentiated
12	Matsumoto [14]	2020	47/M	28	510	Right	Well-differentiated
13	Lieto [15]	2022	61/M	70	N/A	Left	Well-differentiated
14	Cheng [16]	2023	55/M	32	4500	Right	Pleomorphic
15	Our case		55/M	47	7500	Left	Well-differentiated

*No.* number, *N/A* not available

Table 2 offers a comprehensive overview of the initial diagnoses, treatments, and outcomes from the 15 documented cases. Among these cases, seven were diagnosed as inguinal hernias, subsequently undergoing inguinal hernia repair procedures. Significantly, two laparoscopic inguinal hernia repair cases led to intraoperative identification of retroperitoneal liposarcoma, prompting immediate cessation of hernia repair and transition to radical surgery. In five instances, an anterior open technique was employed for inguinal hernia repair; however, postoperative diagnoses of retroperitoneal liposarcoma necessitated re-operation. The authors highlight challenges in the intraoperative diagnosis of retroperitoneal liposarcoma during inguinal hernia repair using anterior open techniques. Postoperative recurrence has been documented in two cases involving re-operations following initial hernia repair. Leão et al. chronicled described a patient who, after refusing routine follow-up, exhibited recurrence nine years postoperatively. The patient subsequently underwent tumor resection, but unfortunately passed away postoperatively [9]. Similarly, Tardur et al. reported recurrence five months postoperatively, followed by tumor resection and a subsequent 20-month period without recurrence [11]. Curative surgical resection remains the optimal approach for liposarcoma treatment. In the present case, the inguinal hernia stemmed from retroperitoneal liposarcoma. Even within instances of hernia repair, long-term survival, and radical intervention are attainable when accurate postoperative diagnosis guides a subsequent radical tumor resection through re-operation. Cheng et al. presented a case wherein tumor resection followed neoadjuvant radiotherapy, yielding positive margins. Subsequent adjuvant chemotherapy resulted in no recurrence at the 26-month postoperative mark [16].

**Table 2:** Initial diagnosis, treatment methods, and results of the 15 cases

No.	Author	Initial diagnosis	Treatment methods	Outcome	DFS (month)	OS (month)
1	Noguchi [4]	Hernia	Hernia operation → reoperation for tumor resection	Non-recurrence	57	57
2	Mizuno [5]	Tumor	Tumor resection	Non-recurrence	4	4
3	Baldassarre [6]	Hernia	Hernia operation → reoperation for tumor resection	N/A	N/A	N/A
4	Ghimire [7]	Tumor	Tumor resection	Non-recurrence	N/A	N/A
5	Bhandarwar [8]	Tumor	Tumor resection	Non-recurrence	24	24
6	Leão [9]	Hernia	Hernia operation → reoperation for tumor resection	Recurrence	N/A	108
7	McKinley [10]	Tumor	Tumor resection	Non-recurrence	30	30
8	Tardu [11]	Hernia	Hernia operation → reoperation for tumor resection	Recurrence	5	20
9	Fiaschetti [12]	Tumor	Tumor resection	N/A	N/A	N/A
10	Fiaschetti [12]	Tumor	Tumor resection	N/A	N/A	N/A
11	Lechner [13]	Hernia	Laparoscopic exploratory laparotomy → tumor resection	Non-recurrence	22	22
12	Matsumoto [14]	Hernia	Laparoscopic exploratory laparotomy → tumor resection	Non-recurrence	9	9
13	Lieto [15]	Tumor	Tumor resection	Non-recurrence	12	12
14	Cheng [16]	Tumor	Neoadjuvant radiotherapy → tumor resection → Adjuvant chemotherapy	Non-recurrence	26	26
15	Our case	Hernia	Hernia operation → reoperation for tumor resection	Non-recurrence	30	30

*DFS* disease-free survival, *OS* overall survival, *N/A* not available

When excising large retroperitoneal liposarcomas prolapsing through the inguinal canal, the inguinal repair is often necessary. However, a question arises regarding the appropriateness of using artificial materials such as mesh during surgery for malignant tumors. Among the 15 cases listed in Table 2, inguinal repair was performed in 8 cases, with mesh being used for repair in only 2 cases, including our case. Although International guidelines for groin hernia management [19] do not specifically address simultaneous surgery for malignant tumors and inguinal hernia repair using mesh, the safety of simultaneous surgery for malignant tumors, such as prostate cancer, and inguinal hernia repair with mesh has been reported in multiple studies [20–22]. In the two cases that experienced recurrence in our study, inguinal repair was performed, but mesh was not used, and tumor residue was considered the cause of recurrence. Based on these findings, we believe that the use of mesh for repair is acceptable when achieving R0 resection. Additionally, recent reports suggest that repair with mesh may also be acceptable during inguinal hernia repair following bowel resection, provided there is no contamination of the inguinal region. However, if the inguinal region is contaminated, we recommend choosing either repair using tissue suturing or staged repair using mesh.

While a preoperative confirmation of the diagnosis is ideal, specific circumstances, such as hernia incarceration, might prompt the need for inguinal hernia repair. In laparoscopic surgery, encountering adipose tissue protrusion instead of an inguinal hernia necessitates an immediate decision to halt the procedure and initiate a comprehensive systemic assessment. Conversely, the anterior open technique poses challenges for intraoperative diagnosis. Consequently, surgically excised adipose tissue should be treated as a spermatic cord lipoma, prompting the need for pathological examination.

## Conclusions

While spermatic cord lipomas are frequently encountered intraoperatively during inguinal hernia repairs, it is imperative to acknowledge the potential for retroperitoneal liposarcomas to prolapse through the inguinal canal. The challenge of intraoperative diagnosis becomes particularly pronounced when utilizing the anterior open technique, which lacks the advantage of preoperative diagnosis. It is important that intraoperative fat tissue is sent to pathology. If a retroperitoneal liposarcoma is diagnosed postoperatively and a complete resection can be achieved at reoperation, a long-term prognosis can be expected. Furthermore, it is worth noting that repair of the inguinal region may involve the use of mesh, a practice that should be considered permissible in appropriate cases.

In this case, the previous inguinal hernia likely originated from the prolapse of a retroperitoneal liposarcoma. Therefore, recommending a preoperative evaluation, including a CT scan, is prudent. This approach gains significance, as the presence of a fatty mass within the hernia serves as an indicator that warrants consideration of a possible retroperitoneal lipoma or tumor.

## Data Availability

The data supporting the findings of this study are available within the article.
